# Longer-Term Omega-3 LCPUFA More Effective Adjunct Therapy for Tuberculosis Than Ibuprofen in a C3HeB/FeJ Tuberculosis Mouse Model

**DOI:** 10.3389/fimmu.2021.659943

**Published:** 2021-04-28

**Authors:** Frank E. A. Hayford, Mumin Ozturk, Robin C. Dolman, Renee Blaauw, Arista Nienaber, Du Toit Loots, Frank Brombacher, Cornelius M. Smuts, Suraj P. Parihar, Linda Malan

**Affiliations:** ^1^ Centre of Excellence for Nutrition, North-West University, Potchefstroom, South Africa; ^2^ Department of Dietetics, School of Biomedical and Allied Health Sciences, College of Health Sciences, University of Ghana, Accra, Ghana; ^3^ International Centre for Genetic Engineering and Biotechnology (ICGEB), Cape Town-Component, University of Cape Town, Cape Town, South Africa; ^4^ Institute of Infectious Diseases and Molecular Medicine (IDM), Division of Immunology and South African Medical Research Council (SAMRC) Immunology of Infectious Diseases, University of Cape Town, Cape Town, South Africa; ^5^ Division of Human Nutrition, Stellenbosch University, Tygerberg, Cape Town, South Africa; ^6^ Laboratory of Infectious Disease Metabolomics, Human Metabolomics, North-West University, Potchefstroom, South Africa; ^7^ Wellcome Centre for Infectious Diseases Research in Africa (CIDRI-Africa) and Institute of Infectious Diseases and Molecular Medicine (IDM), University of Cape Town, Cape Town, South Africa; ^8^ Division of Medical Microbiology, Institute of Infectious Diseases and Molecular Medicine (IDM), Department of Pathology, Faculty of Health Sciences, University of Cape Town, Cape Town, South Africa

**Keywords:** ibuprofen, C3HeB/FeJ mice, tuberculosis adjunctive treatment, repurposed drugs, pharmaconutrition, host-directed therapies, n-3 LCPUFAs

## Abstract

Advancement in the understanding of inflammation regulation during tuberculosis (TB) treatment has led to novel therapeutic approaches being proposed. The use of immune mediators like anti-inflammatory and pro-resolving molecules for such, merits attention. Drug repurposing is a widely used strategy that seeks to identify new targets to treat or manage diseases. The widely explored nonsteroidal anti-inflammatory drug (NSAID) ibuprofen and a more recently explored pharmaconutrition therapy using omega-3 long-chain polyunsaturated fatty acids (n-3 LCPUFAs), have the potential to modulate the immune system and are thus considered potential repurposed drugs in this context. These approaches may be beneficial as supportive therapy to the already existing treatment regimen to improve clinical outcomes. Here, we applied adjunct ibuprofen and n-3 LCPUFA therapy, respectively, with standard anti-TB treatment, in a C3HeB/FeJ murine model of TB. Bacterial loads, lung pathology, lung cytokines/chemokines and lung lipid mediators were measured as outcomes. Lung bacterial load on day 14 post-treatment (PT) was lower in the n-3 LCPUFA, compared to the ibuprofen group (*p* = 0.039), but was higher in the ibuprofen group than the treated control group (*p* = 0.0315). Treated control and ibuprofen groups had more free alveolar space initially as compared to the n-3 LCPUFA group (4 days PT, *p*= 0.0114 and *p*= 0.002, respectively); however, significantly more alveolar space was present in the n-3 LCPUFA group as compared to the ibuprofen group by end of treatment (14 days PT, *p* = 0.035). Interleukin 6 (IL-6) was lower in the ibuprofen group as compared to the treated control, EPA/DHA and untreated control groups at 4 days PT (*p* = 0.019, *p* = 0.019 and *p* = 0.002, respectively). Importantly, pro-resolving EPA derived 9-HEPE, 11-HEPE, 12-HEPE and 18-HEPE lipid mediators (LMs) were significantly higher in the EPA/DHA group as compared to the ibuprofen and treated control groups. This suggests that n-3 LCPUFAs do improve pro-resolving and anti-inflammatory properties in TB, and it may be safe and effective to co-administer as adjunct therapy with standard TB treatment, particularly longer-term. Also, our results show host benefits upon short-term co-administration of ibuprofen, but not throughout the entire TB treatment course.

## Introduction

Tuberculosis (TB) was the leading cause of death from a single infectious agent worldwide until the 2019 outbreak of the Covid-19 disease, surpassing human immunodeficiency virus (HIV)/acquired immunodeficiency syndrome (AIDS) in 2018 (1). Tuberculosis causes an inflammatory reaction that damages the surrounding tissue, causing significant morbidity (2, 3). Host cellular immune responses are exploited (4) to promote the accumulation of permissive phagocytic cells while delaying activation of the acquired responses in TB (5, 6). Thus, therapy targeted towards modulating host inflammatory pathways to reduce abnormal or unwarranted inflammation and lung tissue destruction is a plausible host-directed intervention in TB treatment (7–9). Host-directed intervention administered in conjunction with conventional TB treatment can aid in tissue damage repair, preserve lung function, enhance the effectiveness of TB drug therapy and shorten treatment duration (9).

Recent animal model work suggests that the manipulation of host modulators with repurposed drugs, including pharmaconutrients, may lead to the improvement of TB outcomes associated with common TB morbidities (7, 10–12). Due to the limited advancement in novel TB drug development for more than 40 years now, except for the recent approval of bedaquiline, delamanid and pretomanid (13–15), the use of repurposed drugs to augment current TB therapy has gained much interest. This has led to an interest in exploring the use of ibuprofen as a repurposed drug, and more recently also long-chain omega-3 polyunsaturated fatty acids (n-3 LCPUFAs) as possible adjunct therapies in TB treatment (16–18). Ancillary treatments for diseases like TB associated with inflammation, using repurposed drugs, may ameliorate morbidity and possibly reduce mortality, and are likely to have additionally significant economic, social and survival benefits. Therefore, immune modulating drugs and pharmaconutrients are potentially important agents worth investigating to offer novel host adjunct therapies in TB management (19, 20).

Ibuprofen, a nonsteroidal anti-inflammatory drug (NSAID), inhibits both COX 1 and 2, which metabolise both pro- and anti-inflammatory mediators. This drug, in the absence of TB drug treatment, reduced the percentage of affected lung area, bacillary load and granuloma formation, and increased survival in a C3HeB/FeJ mouse TB model (10, 21). Ibuprofen has also been shown to have no detrimental interaction with either rifampicin (R) or isoniazid (H), neither when used concomitantly with rifampicin, isoniazid, pyrazinamide and ethambutol (RHZE) during anti-tuberculosis therapy in mice (22, 23). However, long-term use of ibuprofen is associated with certain gastrointestinal, low risk cardiovascular, renal and hepatic adverse effects, although these side effects are associated with dose, associated medications, and the patient population (24, 25). Nevertheless, ibuprofen is still considered to have a relatively favourable safety profile among NSAIDs (24).

A possible safer anti-inflammatory or pro-resolving alternative to ibuprofen may be n-3 LCPUFAs. Long-chain n-3 PUFAs are present in oily fish and supplements in the form of EPA and DHA. Their ability to downregulate several aspects of inflammation suggests that these fatty acids might be important in controlling the development and severity of inflammatory diseases, and subsequently, have possible use as a component of a novel therapy approach to various diseases (26). We previously demonstrated that EPA/DHA supplementation lowered systemic and lung inflammation, and decreased lung bacterial burden in C3HeB/FeJ mice infected with *Mycobacterium tuberculosis (Mtb)* in the absence of standard TB treatment (17). However, there is currently no evidence on the interaction of a therapeutic dose of n-3 LCPUFAs with the first-line of anti-TB drugs.

In the present study, we hypothesised that co-administration of ibuprofen or n-3 LCPUFAs, together with standard TB antibiotics, are safe and would potentially improve the clinical outcome and pathology associated with TB. To this end, we investigated the effects of EPA/DHA and ibuprofen, administered as an adjunct to the standard antibiotic treatment-regimen in *Mtb*-infected C3HeB/FeJ mice. Our study is the first to have administered ibuprofen and n-3 LCPUFAs together with standard TB antibiotics, namely rifampicin, isoniazid, pyrazinamide and ethambutol, in the intensive phase, and also in conjunction with rifampicin and isoniazid in the continuation phase in a murine model, in line with TB treatment in humans. We found that EPA/DHA and ibuprofen as adjunct therapy reduce *Mtb* burden and suppress pulmonary immunopathology associated with TB, as compared to untreated controls, and may have the potential to limit TB associated inflammation and improve other clinical outcomes.

## Materials and Methods

### Experimental Design and Dietary Conditioning of Animals

**Figure 1:**
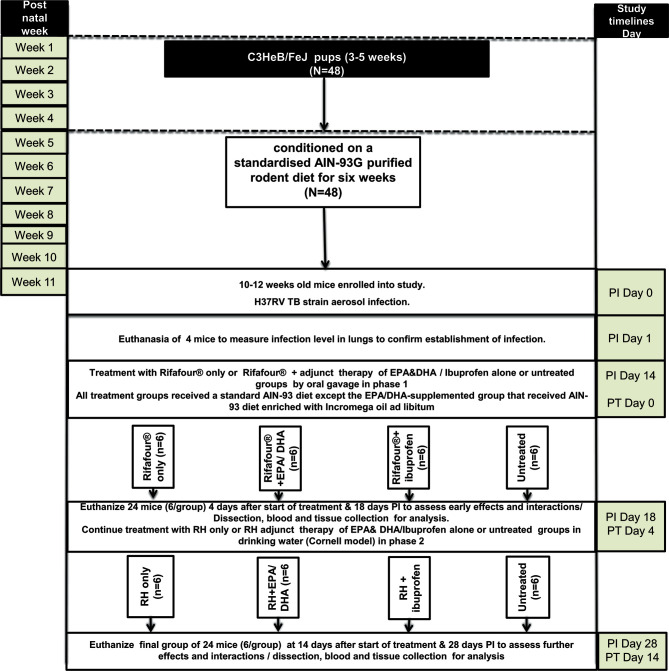
Experimental design of the study. EPA, eicosapentaenoic acid; DHA, docosahexaenoic acid; Rifafour^®^, 150 mg rifampicin + 75 mg isoniazid + 400 mg pyrazinamide + 275 mg ethambutol; RH, rifampicin and isoniazid; N, number of mice; PT: post treatment; PI: post infection; AIN-93G formulation containing soybean oil at 70 g/kg diet and hydrogenated coconut oil at 30 g/kg diet and 8 g Fe/kg (40 ppm Fe); EPA/DHA supplemented diet contains soybean oil at 70 g/kg diet, coconut oil at 27 g/kg diet, Incromega TG4030 oil DHA 500 TG SR (minimum 44% of FA as EPA; minimum 28% of FA as DHA); untreated group received no treatment throughout the entire duration of the experiment.

Forty-eight mice, aged 10-12 weeks, were conditioned on a standardised AIN-93G purified rodent diet for six weeks before infection. The mice were then infected with *Mtb* (50-70 CFU) by means of aerosol inhalation. Two weeks post-infection (PI), the mice were randomly allocated to the four treatment groups: 1) the treated control group received Rifafour^®^ for 3 days (n = 12), followed by rifampicin and isoniazid for 11 days (n = 6); 2); the EPA/DHA group received an EPA/DHA-enriched diet plus Rifafour^®^ for 3 days (n = 12), followed by the EPA/DHA-enriched diet plus rifampicin and isoniazid for 11 days (n = 6); 3); the ibuprofen group received ibuprofen plus Rifafour^®^ for 3 days (n = 12), followed by ibuprofen plus rifampicin and isoniazid for 11 days (n = 6); and 4) the untreated control group received no treatment throughout the entire duration of the experiment (n = 12). Treatment was administered in two phases. In the first phase, ibuprofen and Rifafour^®^ (150 mg rifampicin + 75 mg isoniazid + 400 mg pyrazinamide + 275 mg ethambutol) were administered by oral gavage, and in the second phase, ibuprofen as well as rifampicin and isoniazid (RH) were administered in drinking water (Cornell model). Treatment duration of phase one lasted for three days, representing the initial 2 months intensive phase TB treatment equivalent in humans with the same four medications, Rifafour^®^. Whilst phase two continued for another 11 days, representing the four months less intensive TB treatment phase (continuous phase) humans equivalence with two medications RH (27, 28). Each group consisted of 6 mice, in two independent experiments. All treatment groups received a standard AIN-93 diet; with the exception of the EPA/DHA-supplemented group that received the AIN-93 diet enriched with Incromega oil *ad libitum* as shown in **Figure 1** *(See* **Supplementary File 1** *for the detailed description of how the experiment was conducted).*

### Experimental Mice Model and Ethics Statement

Male and female C3HeB/FeJ mice (obtained from Jackson Laboratory, Bar Harbor, ME), between 10 and 12 weeks of age, were randomly placed in groups of six in a standard type 2 long individually ventilated cage with filter tops, transparent red plastic mouse houses, dried wood shavings and shredded filter paper as floor coverings after infection. Mice were housed in a biosafety level III facility and were exposed to a temperature range set at 22 to 24°C and 12-to-12 hour light cycles. The study was approved by the AnimCare Animal Research Ethics Committee of North-West University, South Africa (ethics number: NWU-00055-19-S5), and the Animal Research Ethics Committee of the University of Cape Town, South Africa (ethics number: FHS AEC 019-023).

### Aerosol Infection

The virulent *Mtb* H37Rv strain was cultured and stocks were prepared and stored at -80°C, as previously described (29). Mice were infected by nebulising with 6 ml of a suspension that contained 2.4 x 10^7^ live bacteria in an inhalation exposure system (model A4224, Glas-Col) for 40 minutes. Four mice were euthanized to confirm the infection dose a day after infection, with each mouse being infected with around 50-70 *Mtb* colony-forming units (CFU).

### Treatment Administration

All drugs used for treatment were either dissolved or suspended in distilled water and administered either by oesophageal gavage or in the drinking water. The following doses were used: in phase one (intensive phase), each mouse received 0.2 mL of antibiotic consisting of Rifafour^®^ (150 mg rifampicin + 75 mg isoniazid + 400 mg pyrazinamide + 275 mg ethambutol) dissolved in 30 ml distilled water through oral gavage administration. In phase two (continuation phase), isoniazid (0.1g/L) and rifampicin (0.1g/L) were delivered to mice in drinking water (30). For the adjunct ibuprofen group, 0.05 g/L of ibuprofen (Nurofen^®^ cherry flavour, purchased from local pharmacy) was administered *ad libitum* in phases one and two. Because of the bitter taste of rifampicin-isoniazid together with ibuprofen, 1% sucrose was added to drinking water for three groups in phase 2. The water consumption was measured in phase two to confirm equal drug intake in all three groups.

### Blood and Tissue Collection

At the end of day 4 and 14 post-treatment, mice were euthanized by exposure to halothane, after which blood was collected *via* cardiac puncture. The blood was collected into EDTA-coated Microtainer^®^ tubes (K2EDTA, 1000 µl, Becton Dickinson), then centrifuged at 8000 rpm. The peripheral blood mononuclear cells (PBMC) were collected from buffy coats and used for fatty acid (FA) analysis. The liver and lung lobes were removed aseptically and weighed before preparation. The left lung lobe was homogenized in saline and 0.04% Tween-80 for the analysis of the bacillary load and lung cytokines. The right superior and post-caval lung lobes were snap-frozen in liquid nitrogen and stored at -80°C for lung LM analyses. The right middle lobe was fixed in 10% neutral buffered formalin for histological analysis.

### Bacteria Loads and Lung Histopathology

Lung lobes and spleen were aseptically removed, homogenized and serial dilutions prepared. Dilutions were plated onto Difco™ Middlebrook 7H10 Agar medium (BD Biosciences, Johannesburg, South Africa), with oleic acid-albumin-dextrose-catalase (OADC) supplementation and 0.5% glycerol. The CFU were counted 21 days after incubation at 37°C. The results were expressed as log_10_CFU. For the assessment of lung pathology, the right middle lobes of the lungs were fixed in 10% buffered formalin and embedded in paraffin wax after processing in Leica TP 1020 Processor for 24 hours. 3-µm-thick sections (three sections with 30 µm-apart) of the embedded tissues were cut and stained with hematoxylin-eosin (H&E) stain. The images were acquired in Nikon Eclipse 90i microscope and analysed with NIS-Elements AR software (Nikon Corporation, Tokyo, Japan) to determine the granulomatous area and free alveolar space as a percentage of the total lung tissue (31).

### Determination of Total Phospholipid Fatty Acid Composition

Total phospholipid FA composition was analysed by gas chromatography-tandem mass spectrometry as previously described (17). Fatty acids were extracted from ~200 µL PBMC with chloroform:methanol (2:1, v:v; containing 0.01% BHT) by a modification of the method of Folch et al. (32). The composition of EPA (20:5n-3), DHA (22:6n-3), arachidonic acid (AA, 20:4n-6), osbond acid (22:5n-6), total n-3 LCPUFA, total omega-6 long-chain polyunsaturated fatty acid (n-6 LCPUFA) and total n-6/n-3 LCPUFA ratio as a percentage of total phospholipid fatty acids were determined.

### Extraction and Quantification of Lipid Mediators

Lipid mediators in crude lung homogenates were extracted and analysed by liquid chromatography-tandem mass spectrometry. Lipid mediators were extracted from lung tissue, in 10 µl/mg homogenization buffer (phosphate-buffered saline), with solid-phase extraction using Strata-X (Phenomenex, Torrance, CA). The method was modified for Strata-XSPE columns from a previously described method (33). Data were quantified with Masshunter B0502, using external calibration for each compound and internal standards [PGD2-d4, PGE2-d4, PGF2-d4 and 5- and 12-HETE-d8; 1000 pg of each (Cayman Chemicals, Ann Arbor, MI)] to correct for losses and matrix effects. Extracted and quantified LMs included: DHA-derived pro-resolving 17-hydroxydocosahexaenoic acid (17-HDHA) and protectin D1 (PD1); EPA-derived pro-resolving LM intermediates 2-, 5-, 9-, 11-, 15- and 18-hydroxyeicosapentaenoic acid (HEPE); AA-derived pro-inflammatory intermediates 5-, 8-, 9-, 11-, 12- and 15-hydroxyeicosatetraenoic acid (HETE); AA-derived prostaglandin D1 (PGD1), prostaglandin E2 (PGE2), prostaglandin F2α (PGFα2); and thromboxane B2 (TBXB2).

### Measurement of Cytokine/Chemokine in Lung Homogenates

Lung homogenates were centrifuged at 3000g and supernatants were stored at -80°C until further analysis. Interleukin (IL)-1α, IL-1β, IL-2, IL-3, IL-4, IL-5, IL-6, IL-10, IL-12, IL-17, monocyte chemoattractant protein 1 (MCP-1), interferon-gamma (IFN-γ), tumor necrosis factor-alpha (TNF-α), granulocyte-macrophage colony-stimulating factor (GM-CSF), macrophage inflammatory protein 1-alpha (MIP-1 α) and regulated on activation normal T-cell expressed and secreted (RANTES) levels were measured using the Quansys Biosciences Q-Plex™ Mouse Cytokine Screen (West Logan, UT, USA) 16-plex array for mouse cytokines according to manufacturer’s instructions. Arrays were analyse using the Q-View Imager Pro and Q-View Software.

### Statistical Analyses and Data Representation

Statistical analyses were computed using IBM SPSS Statistics software version 23 and GraphPad Prism Software version 8.2 (GraphPad Software Inc., La Jolla, CA, USA). A minimum sample size of 6 per treatment group was calculated for a two-sided alpha of 0.05 and a power of 80% as previously described (34). The normality of the data was evaluated by histogram visual inspection and Kolmogorov-Smirnov test. All data are presented as mean ± standard error of the mean (SEM). Treatment effects were examined using one-way analysis of variance (ANOVA) and the Tukey post-hoc test. Statistically significant differences are designated as follows: *p < 0.05; **p < 0.01; ***p < 0.001.

## Results

### Ibuprofen Adversely Affected Bactericidal Efficacy of TB Drugs, However, EPA/DHA Did Not

**Figure 2:**
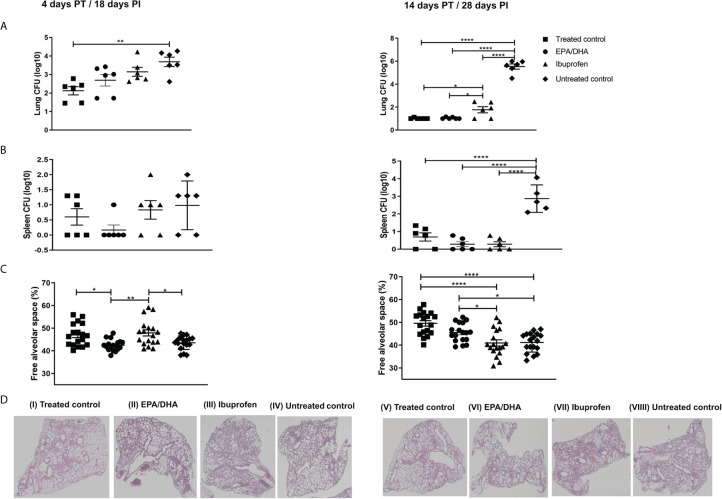
Effect of adjunctive treatment on lung, spleen bacillary load and lung histopathology. **(A)** Lung bacillary load, **(B)** spleen bacillary load, **(C)** percentage of free alveolar space, and **(D)** haematoxylin-eosin stained sections of the lungs of selected representative group, after 18 [D(I-IV)] and 28[D(V-VIII)] days PI treatment period. All mice except untreated controls were on standard TB antibiotics Rifafour^®^ for 4 days of treatment, then rifampicin and isoniazid (RH) for 10 days. The data represented as mean ± SEM of n=6 mice/group and representative of two independent experiments. One-way ANOVA followed by the Tukey post-hoc test was used to compare means, significance at *P < 0.05, **P < 0.01, ****P < 0.0001. CFU, colony-forming units; EPA, eicosapentaenoic acid; DHA, docosahexaenoic acid PT, post-treatment; PI, post-infection.

We investigated whether EPA/DHA increased the efficacy of TB drugs. Mice were infected with *Mtb* for 2 weeks and then administered with TB drugs to euthanize at 4 and 14 days post-treatment (**Figure 2**). Bacillary load in lung and spleen was determined to ascertain whether EPA/DHA or ibuprofen co-administration interfered with the standard TB treatment, Rifafour and RH. Lung and spleen bacterial burdens were similar between standard TB treatment and adjunct EPA/DHA treatment groups (**Figures 2A, B**). Interestingly, lung bacterial loads were lower on day 14 PT in the EPA/DHA group, compared to the ibuprofen group (p = 0.039). This suggests that it may be safe to co-administer EPA/DHA as adjunct therapy with standard TB treatment, particularly in the longer term. However, lung burdens were higher in the adjunct ibuprofen group, as compared to the standard treatment control group on day 14 PT (*p* = 0.032), with a similar trend on 4 PT (*p* = 0.051), suggesting it may not be suitable to co-administer ibuprofen during standard TB treatment with regards to lung bacterial burdens. However, spleen burden was lower in the ibuprofen group, compared to the other treatment groups, although not significantly. Both lung and spleen burdens in the non-treated controls remained higher during the infection, as expected (**Figures 2A, B**).

### Ibuprofen and EPA/DHA as an Adjunct Therapy Improved Lung Pathology

Lung histology sections were stained with H&E to determine tissue pathology and quantitate the percentage of free alveolar space. We found significantly more free alveolar space in the treated control and ibuprofen groups than in the EPA/DHA group at the initial stage (day 4 PT; *p* = 0.011 and *p* = 0.002, respectively), but significantly more alveolar space in the EPA/DHA and the treated control groups than in the ibuprofen group after 14 days of treatment (*p* = 0.035 and *p <*0.001, respectively). This indicates that adjunctive ibuprofen may be beneficial in the early phase, whereas EPA/DHA may be more beneficial in the longer term, considering the improvement to tissue pathology. As expected, the untreated control group had significantly less free alveolar space than the treated groups throughout entire course of the study (**Figures 2C, D**).

### Dietary EPA/DHA Supplementation and Ibuprofen Treatment Altered Lung Inflammatory Cytokines During Co-Administration With Standard TB Therapy

**Figure 3:**
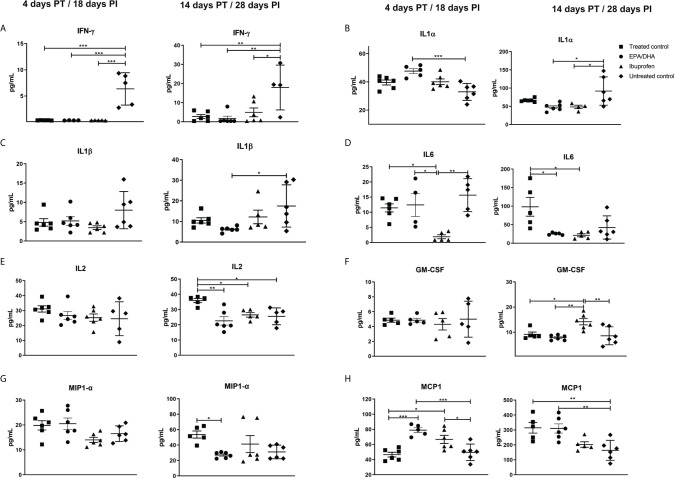
Treatment effects on lung cytokine/chemokines levels. **(A)** IFN-γ, **(B)** IL-1α, **(C)** IL-1β, **(D)** IL6, **(E)** IL2, **(F)** GM-CSF, **(G)** MIP-1 α and **(H)** MCP-1. All data are presented in pg/mL. All mice except untreated controls were on standard TB antibiotics Rifafour^®^ for 4 days of treatment, then rifampicin and isoniazid (RH) for 10 days. All values represent mean ± SEM. Results repeated in two experiments, data shown for one experiment (n = 6 per group). One-way ANOVA followed by Tukey’s post-hoc test was used to compare means, *P < 0.05, **P < 0.01, ***P < 0.001. EPA, eicosapentaenoic acid; DHA, docosahexaenoic acid; IL, interleukin; IFN- γ, interferon γ; IL-1α, interleukin 1 alpha; IL-1β, Interleukin 1 beta, GM-CSF, granulocyte-macrophage colony-stimulating factor; MIP-1 α, macrophage inflammatory protein 1-alpha and MCP-1, Monocyte chemoattractant protein-1; PT, post-treatment; PI, post-infection.

The concentrations of cytokines and chemokines in the lung homogenates were measured to assess local inflammatory effects. Cytokines/chemokines are responsible for recruitment of polymorphonuclear cells (PMN), monocytes and lymphocytes, which are involved in inflammatory reactions, mediate adaptive immune cell responses, act as anti-inflammatory agents to resolve inflammation and limit tissue damage during *Mtb* infection (35). Interferon γ (IFN-γ) was significantly lower in all the treated groups at 4 days (treated control, *p* < 0.001; EPA/DHA, *p* =0.003 and ibuprofen, *p* < 0.001) and 14 days (treated control, *p* = 0.005; EPA/DHA, *p* =0.003 and ibuprofen, *p* = 0.017) PT, compared to the untreated control mice (**Figure 3A**). Interleukin 1 alpha (IL-1α) was significantly higher in the EPA/DHA group at 4 days PT (*p*= 0.001) and lower in the EPA/DHA and ibuprofen groups at 14 days PT (*p*= 0.012 and *p*= 0.028, respectively), compared to the untreated control mice (**Figure 3B**). Interleukin 1 beta (IL-1β) was significantly lower in the EPA/DHA group at 14 days PT (*p*= 0.033), compared to the untreated control mice, while no difference was observed at the earlier time point (**Figure 3C**). Interleukin 6 (IL-6) was lower in the ibuprofen group at 4 days PT, compared to the treated control, EPA/DHA and untreated control groups (*p* = 0.019, *p* = 0.019 and *p* = 0.002 respectively). Subsequently, IL6 was lower in both the EPA/DHA and ibuprofen groups by day 14 PT than in the treated control group (*p* = 0.012 and *p* = 0.012, respectively, **Figure 3D**). Concentrations of IL2 were lower in the EPA/DHA and ibuprofen groups than in the treated control group by day 14 PT (*p* = 0.002 and *p* = 0.039, respectively, **Figure 3E**). This suggests that the effect of adjunct EPA/DHA administration on the lung inflammatory cytokine profile was more profound at the later phase of treatment consistent with lower lung burdens, whereas, ibuprofen adjunct treatment decreased inflammatory cytokines in the early phase of treatment.

Granulocyte-macrophage colony stimulating factor (GM-CSF) was lower in the EPA/DHA group, compared to the ibuprofen group by day 14 PT (*p* = 0.003) and higher in the ibuprofen group than in the treated controls (*p* = 0.025, **Figure 3F**), which suggests an interesting regulation between GM-CSF secretion and COX1/COX2 inhibition. Macrophage inflammatory protein 1-alpha (MIP1-α) was lower in the EPA/DHA group by day 14 PT (*p* = 0.048, **Figure 3G**). Monocyte chemoattractant protein-1(MCP1) was higher in the EPA/DHA and ibuprofen groups, compared to the treated controls at day 4 PI (*p* = 0.002 and 0.012, respectively), and higher in the treated controls and EPA/DHA group than in the untreated controls by day 14 PT (*p* = 0.009 and 0.008 respectively, **Figure 3H**). These results support varied effects of ibuprofen and EPA/DHA adjunct treatment on inflammatory chemokines.

Interleukin-5 (IL5) was higher in the treated controls and ibuprofen group than untreated controls on days 4 PT (*p* = 0.034 and *p* = 0.016, respectively, **Supplementary Figure 1A**). Interleukin-10 (IL-10) was lower in the ibuprofen than in the treated control (*p* = 0.044, **Supplementary Figure 1B**), while IL-12 was lower in the EPA/DHA group than in the untreated controls (*p* = 0.023, **Supplementary Figure 1C**) both at day 14 PT. Interleukin-4 (IL-4), IL-17, TNF-α, IL-3 and RANTES all showed lower trends in the EPA/DHA treatment groups than in the other treatment groups by day 14 PT, although not significantly (**Supplementary Figures 1D–H**).

### Effects of Treatments on PBMC Fatty Acid Composition

**Table 1** shows the phospholipid FA composition of PBMC of four groups of mice measured at 4 and 14 days PT. The change in membrane phospholipid FA composition of immune cells plays a vital role in immune and inflammatory responses (16). The EPA/DHA group had a significantly higher EPA composition than all other groups (day 4 PT, all *p* < 0.001 and day 14 PT, all *p* < 0.001), while DHA was higher in the ibuprofen group than in the untreated controls at day 18 PI (*p* = 0.007). The composition of the total n-3 LCPUFA in the EPA/DHA treatment group was lower than that of the untreated controls (day 4 PT, *p* = 0.003), but was higher in the EPA/DHA treatment group after 14 days of treatment (*p* = 0.039). Regarding n-6 PUFAs, the ibuprofen treatment group had higher total n-6 LCPUFAs (day 4 PT, *p* = 0.052 and day 14 PT, *p* < 0.001) and total n-6/n-3 LCPUFA ratio (day 4 PT, *p* = 0.009 and day 14 PT, *p* = 0.001) compositions, compared to the EPA/DHA treatment groups over the course of infection at different time points. These trends were similar for the AA (day 4 PT, *p*=0.006 and day 14 PT, *p* < 0.001) and osbond acid (day 4 PT, *p* = 0.19 and day 14 PT, *p* = 0.003) compositions, respectively. Consistent with n-3 levels, the effects of EPA/DHA treatment on n-6 levels were more profound on day 28 PI. The higher levels of pro-resolving and anti-inflammatory FAs, such as the n-3 LCPUFAs, present in the membrane phospholipid of the EPA/DHA treatment group, together with the reduced amounts of pro-inflammatory FAs, such as AA, noted in later time points, suggests the EPA/DHA adjunct treatment in TB may be more effective than ibuprofen co-treatment in terms of decreasing exaggerated inflammation.

**Table 1:** Phospholipid fatty acid composition of PBMC in *Mtb-*infected C3HeB/FeJ mice receiving EPA/DHA or ibuprofen adjunct treatment at different time points^#^.

4 days post-treatment					
Fatty acids	Rifafour	Rifafour + EPA/DHA	Rifafour + ibuprofen	untreated control	P- value
**20:5n-3 (EPA)**	0.40 ± 0.02^b^	0.60 ± 0.02^a^	0.35 ± 0.01^b^	0.41 ± 0.02^b^	<0.001
**22:6n-3 (DHA)**	11.38 ± 0.32^b^	11.54 ± 0.19^b^	12.00 ± 0.20^a^	12.67 ± 0.21^a,b^	0.007
**Total n-3 LCPUFA**	12.56 ± 0.32^b^	12.99 ± 0.17^b^	13.12 ± 0.19^a,b^	14.05 ± 0.22^a^	0.003
**20:4n-6 (AA)**	17.72 ± 0.37^b^	18.38 ± 0.38^a,b^	19.31 ± 0.26^a^	17.83 ± 0.06^b^	0.006
**22:5n-6 (osbond)**	0.93 ± 0.60	0.89 ± 0.04	0.92 ± 0.03	1.03 ± 0.02	0.19
**Total n-6 LCPUFA**	22.22 ± 0.50^b^	23.22 ± 0.46^a,b^	23.82 ± 0.27^a^	22.83 ± 0.16^a,b^	0.052
**Total n-6/n-3 LCPUFA ratio**	1.77 ± 0.05^a^	1.79 ± 0.03^a^	1.82 ± 0.03^a^	1.63 ± 0.02^b^	0.009
**14 days post-treatment**					
	**RH**	**RH + EPA/DHA**	**RH + ibuprofen**	**untreated control**	**P- value**
**20:5n-3 (EPA)**	0.32 ± 0.0^b^	0.74 ± 0.08^a^	0.33 ± 0.00^b^	0.35 ± 0.00^b^	<0.001
**22:6n-3 (DHA)**	11.87 ± 0.12	12.19 ± 0.37	12.50 ± 0.13	12.26 ± 0.21	0.23
**Total n-3 LCPUFA**	13.12 ± 0.11^b^	14.12 ± 0.38^a^	13.90 ± 0.19^a,b^	13.76 ± 0.24^a,b^	0.039
**20:4n-6 (AA)**	18.26 ± 0.13^a^	17.24 ± 0.24^b^	18.83 ± 0.13^a^	18.40 ± 0.08^a^	<0.001
**22:5n-6 (Osbond)**	1.18 ± 0.03^a,b^	1.07 ± 0.03^b^	1.24 ± 0.03^a^	1.27 ± 0.04^a^	0.003
**Total n-6 LCPUFA**	23.69 ± 0.15^b^	22.51 ± 0.22^c^	24.56 ± 0.17^a^	23.99 ± 0.16^a,b^	<0.001
**Total n-6/n-3LCPUFA ratio**	1.81 ± 0.01^a^	1.60 ± 0.05^b^	1.77 ± 0.02^a^	1.75 ± 0.02^a^	0.001

^#^Values are reported as mean ± SEM percentage of total fatty acids from one experiment shown as representative of two independent experiments (n = 6 per group). Means in a row without common superscript letters differ significantly, P < 0.05. One-way ANOVA, with Tukey post hoc test was used to test effects between groups. AA, arachidonic acid; DHA, docosahexaenoic acid; EPA, eicosapentaenoic acid; LCPUFA, long-chain polyunsaturated fatty acids; N, total number of mice used in experiment; PBMC, peripheral blood mononuclear cell; Rifafour, rifampicin 150 mg + isoniazid 75 mg + pyrazinamide 400 mg + ethambutol 275 mg; RH, rifampicin and isoniazid.

### Dietary EPA/DHA Treatment Elevated Pro-Resolving and Reduced Pro-Inflammatory Lung Lipid Mediators, as Compared to Ibuprofen

**Figure 4:**
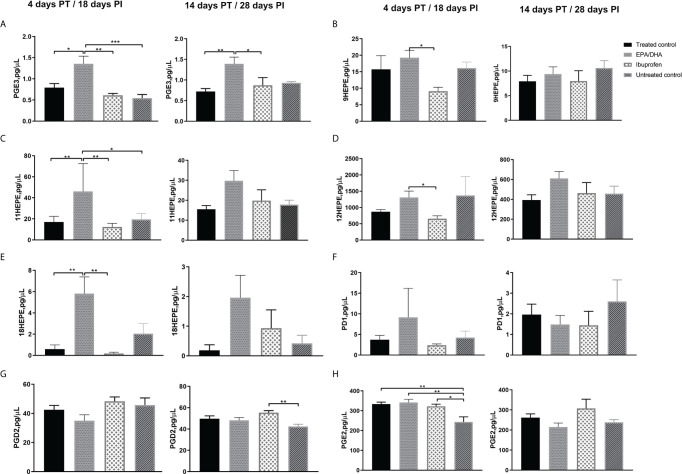
Treatment effects of lipid mediators in crude lung homogenate at the local site of intervention. **(A)** PGE3, **(B)** 9-HEPE, **(C)** 11-HEPE, **(D)** 12-HEPE, **(E)** 18-HEPE, **(F)** PD1, **(G)** PGD2 and **(H)** PGE2. All data are presented in pg/µL. All mice, except untreated controls, were on standard TB antibiotics Rifafour^®^ for 4 days of treatment, then rifampicin and isoniazid (RH) for 10 days. All values represent mean ± SEM. Results repeated in two experiments, data shown for one experiment (n=6 per group). One-way ANOVA followed by Tukey’s post-hoc test was used to compare means, *P < 0.05, **P < 0.01 and ***P < 0.001. EPA, eicosapentaenoic acid; DHA, docosahexaenoic acid; HEPE, hydroxyeicosapentaenoic acid; PGD2, prostaglandin D2; PGE3, Prostaglandin E3; PD1, Protectin D1 and PGE_2_, prostaglandin E_2_; PT, post treatment; PI, post infection.

Crude lung homogenate LMs were measured in order to determine the various treatment effects at the site of disease (the lung). The concentration of EPA-derived PGE3 in the EPA/DHA treated group was comparatively higher than the ibuprofen and the treated control groups on both day 4 PT (*p* = 0.002 and *p* = 0.014, respectively) and day 14 PT (*p* = 0.046 and *p* = 0.008, respectively; **Figure 4A**). Similarly, higher concentrations were observed in the EPA/DHA group for EPA-derived pro-resolving LM intermediates 9-HEPE (day 4 PT, EPA/DHA vs ibuprofen, *p* = 0.017), 11-HEPE (day 4 PT, EPA/DHA vs ibuprofen, *p* = 0.005; EPA/DHA vs treated control, *p* = 0.011 and EPA/DHA vs untreated, *p* = 0.022), 12-HEPE (day 4 PT, EPA/DHA vs ibuprofen, *p* = 0.035), and 18-HEPE (day 4 PT, EPA/DHA vs ibuprofen, *p* = 0.005 and EPA/DHA vs treated control, *p* = 0.006) (**Figures 4B–E**).Trends of higher concentrations of DHA-derived pro-resolving PD1 (**Figure 4F**) and 17HDHA (**Supplementary Figure 2A**) LMs were seen more in the EPA/DHA group than in the other treatment groups, although not significantly.

Pro-inflammatory AA-derived PGD2 was significantly lower in the ibuprofen group than the untreated control group after 14 days of treatment (**Figure 4G**), while PGE2 was significantly higher in all the treatment groups than the untreated control group after 4 days of treatment (**Figure 4H**). No significant differences were seen in the treatment effects of AA-derived pro-inflammatory intermediates 5-, 8-, 9-, 11-, 12- and 15-HEPE among the treatment groups (**Supplementary Figures 2D–H**).

## Discussion

Improved host-directed therapies (HDTs) to augment standard TB treatment has gained attention of late, due to its potential to overcome the obstacles faced by current antibiotic therapies (9). This approach, amongst other mechanisms, aims to improve the host defence mechanisms, and/or modulate excessive inflammation, by altering the host’s response, rather than targeting the *Mtb* itself (36, 37). Recently, much research was done determining the effects of various repurposed drugs as adjunctive agents (4, 38, 39), including n-3 LCPUFAs (17, 40, 41) and ibuprofen (10, 42), with varying results in the field of TB.

In the current investigation we determined that the co-administration of EPA/DHA, together with standard TB antibiotic treatment, did not interfere with the bactericidal effects of antibiotics. Moreover, this adjunct treatment approach reduced the percentage of affected lung area compared to standard therapy after two weeks of treatment. EPA/DHA lowered lung inflammation while increasing the production of pro-resolving LMs. This suggests that it may be safe and effective against tuberculosis to co-administer EPA/DHA as adjunct therapy with standard TB treatment. However, ibuprofen adjunct therapy appears to have attenuated the effect of the TB antibiotics on lung bacterial burden and resulted in a reduced free alveolar space after the initial fourteen days of co-administration, already showing a trend towards the former effect at four days. Nonetheless, ibuprofen did suppress TB lung pathology by decreasing inflammatory cytokines, markedly IL6, in the early phase of treatment which also likely caused alveolar space to be higher in the ibuprofen group compared to the EPA/DHA group at day 4. This suggests that ibuprofen may be beneficial in reducing inflammation, thus preventing TB associated inflammatory pathology in the early phase of treatment. However, considering ibuprofen’s possible interference with TB drug efficacy, its suitability as adjunct long-term treatment seems limited.

Morbidity and mortality in TB are associated with a failure to resolve lung immunopathology, due to the local inflammatory response of the host to ongoing *Mtb* infection (43, 44). This could be resolved with adjunctive therapy by improving the antibiotic efficacy (45). Concerning lung pathology, EPA/DHA together with TB antibiotics in the current study significantly reduced bacteria burden and mitigated excess pulmonary inflammatory damage in C3HeB/FeJ mice when compared to the ibuprofen treatment group, as indicated by the significantly less free alveolar space observed in the lungs of the ibuprofen group, compared to the EPA/DHA and treated control groups after 14 days of treatment. A similar observation was made in our previous work, where EPA/DHA supplementation alone, in the absence of TB antibiotics, resulted in enhanced bactericidal effects and inflammation resolution (17). This could be related to the enhanced phagocytic ability of immune cells *via* special pro-resolving mediators (SPMs): resolvins and protectins (46). These SPMs have been found to stimulate phagocytosis of bacteria, both killing and clearing (47, 48). The inflammation lowering effects observed in our study further demonstrates the beneficial effect of n-3 LCPUFAs as immuno-resolvents (49) with anti-inflammatory properties (50). Earlier murine macrophage-like cell lines and animal models, however, have reported mixed results. Similarly to our findings, 8-week old female BALB/c mice on n-3 LCPUFA-supplemented diet (EPA content 1.5% and DHA 1.1% of total energy), in the absence of TB antibiotics, showed reduced bacterial loads (CFU) in the lung and spleen at 21 post-infection (40). However, on the contrary murine macrophage-like cell lines infected with virulent H37Rv *Mtb* and treated with DHA only, had higher bacterial loads compared with the control group at 3 days post-infection (51). A possible reason for these differences may be due to the one experiment using cell lines; the other was done using an animal model. However, fat-1 transgenic mice, which endogenously produce n-3 PUFAs, infected with virulent H37Rv Mtb *via* the aerosol route, also had increased bacterial loads in the spleen at 4-, 8- and 12-weeks post-infection (41). This observation could partly be explained by diminished activation, recruitment and anti-mycobacterial immune response in the fat-1 mice, resulting in reduced resistance to tuberculosis (52, 53). In our model, dietary supplementation of EPA/DHA slightly shifted the n-6/n-3 ratio towards n-3 LCPUFAs but not as exaggerated as seen in fat-1 transgenic mice (41). Since excess amounts of n-3 LCPUFAs can be unfavourable when treating bacterial infection, more subtle changes by dietary supplementation of EPA/DHA, may serve better for reducing the inflammation and bacterial load.

The results also show that ibuprofen treatment appears to have attenuated the effect of the TB antibiotics in the lungs for the first 14 days of treatment. These findings were in congruence with the findings of Mortensen et al., who observed that treatment with the cyclooxygenase inhibitor (COXi); ibuprofen did not reduce the bacterial burden in the lungs and spleen after aerosol infection in a CB6F1 mouse model. They also observed little or no impact on inflammation (54). The researchers argued that the route of infection, rather than the dose of ibuprofen was a cause for the observed outcome, as a similar dose of *Mtb* Erdman intravenously infected CB6F1 mice, showed a reduced pulmonary bacterial burden, as well as decreased lung infiltration of neutrophils, using the same intervention. Although the administration of ibuprofen alone was shown to reduce the percentage of affected lung area by alleviating excessive inflammation, and also reduce the bacillary load in mice infected with the intravenous route (10), it has also been demonstrated to have no direct bactericidal activity against *Mtb* (22, 42). Notwithstanding, our findings do suggest that ibuprofen does confer some advantage to the host by limiting TB-associated inflammation in the short-term.

The supplementation of n-3 LCPUFAs in this study resulted in a comparatively elevated cell membrane composition of pro-resolving lung LM, most profoundly noticeable at 4 days post treatment (as indicated by elevated concentrations of the less inflammatory EPA-derived PGE3 and EPA-derived pro-resolving LM intermediates: 9-HEPE, 11-HEPE, 12-HEPE and 18-HEPE, compared to the ibuprofen and the treated control groups). Additionally, a decreased trend of the lung pro-inflammatory AA-derived lipid mediators PGE2 and PGF2α in the EPA/DHA treatment, comparative to the treated control and the ibuprofen groups after 14 days (although not statistically significant) was observed. A study by Mancuso et al. reported a similar finding, where fish oil altered pro-resolving lipid mediators in the bronchoalveolar lavage fluids (55). There was also a corresponding significant reduction in the pro-inflammatory cytokine concentrations of IL-2 and IL-6 with EPA/DHA supplementation, as compared to the treated controls. In turn, reduced IL-2 levels can result in lower T cell proliferation and decreased activation of CD8^+^ effector T cells (56). Reduced IL-2 levels can also favour memory T cell formation over effective T cells, which in turn can lead to a dampening of excessive inflammatory responses (57, 58). Similarly, increased levels of IL-6 have been shown to correlate to human disease progression due to its role in inflammation and tissue damage (59, 60). There was a similar observation with the ibuprofen treatment group, where IL-2 and IL-6 were reduced, compared to the treated controls. Worth noting was the significantly reduced concentrations of IL-6 observed in the ibuprofen treatment group at four days post-treatment, compared with the EPA/DHA supplemented group, supporting the previously observed association of ibuprofen treatment on IL-6 levels, as was seen in cystic fibrosis patients (61). Likewise, the pro-inflammatory lipid mediators 5-, 8-, 9-, 12- and 15- HETE were also reduced in ibuprofen treatment after four days, although not significantly (data not shown).

Initially, there was a higher production of DHA-derived pro-resolving 17HDHA and PD1 in the EPA/DHA group, compared to the other treatment groups. This could possibly explain the lowering of the pro-inflammatory mediator PGE_2_ (62) and inhibition of Th1 type cytokine IFN-γ (63). PD1 has demonstrated to be a potent agonist of resolution of inflamed tissues and might have therapeutic potential when sustained inflammation and/or impaired resolution are constituents of pathologic pathways (64). PD1 also blocks airway hyper-responsiveness, counter-regulating signalling in an allergic airway, leading to a possible new therapeutic strategy for modulating inflammation in the asthmatic lung (62).

Monocyte chemotactic protein-1 (MCP-1) was significantly higher with EPA/DHA and ibuprofen treatments, in the initial stages of treatment when compared to the treated controls, and continued to increase over the treatment period. MCP-1 has, however, been shown to recruit and direct leukocyte movement during inflammation, and may have a negative influence on T-cell immunity (65). Furthermore, tuberculosis severity is associated with higher levels of MCP-1, since MCP-1 can induce recruitment of permissive monocytes and *Mtb* localisation to the lung parenchyma (66, 67). However, in the presence of antibiotic pressure, the lung bacterial burden is reduced, which may override any negative impact of elevated MCP-1 levels in the lungs. In a recent study, it has been shown that MCP-1 can also polarise alveolar macrophages to egress from the airway and interact with other immune cells around TB granulomas to gain killing effector functions (68).

Even though most of the inflammatory cytokine profiles were similar in the EPA/DHA group when compared to the other treatment groups, a large change was seen in the pro-resolving LM levels. Hence, our findings support the assertion that n-3 LCPUFAs have both inflammation and pro-resolving properties, and supplementation with this will not inhibit the host’s natural immune and inflammatory responses necessary for protection against *Mtb*. This is in agreement with a study by Serhan et al., which showed that SPMs, which are derivatives of EPA/DHA, are not immunosuppressive and do not block inflammation, but instead produce pro-resolving effects (46). Likewise, the effect of ibuprofen on the inflammatory cytokines IL-6 and IL-2, thus supports its ability to reduce inflammation and resolve host-mediated immune pathology (54, 69) *via* actions of cyclooxygenase (COX) 1 and/or 2 inhibition or modulation.

Our results are further strengthened by the fact that we used a murine model that is already well established and was successfully used before to investigate the interaction between TB lesion pathology and treatment (70). It also reflects human pulmonary TB well, as it forms human-like lesions during *Mtb* infection (71). This model has been previously reported to be a relevant disease model that can be used to explore new TB therapies (70, 71). Furthermore, this study used an experimental design that mimicked the different phases of human TB treatment, i.e. the initial intensive treatment phase, followed by the continuous treatment phase, and was also designed to simulate the acute immune response induced by *Mtb* infection. We additionally analysed local markers of inflammation, and administered the treatment (EPA/DHA and ibuprofen) together with standard TB antibiotics, in order to better understand the possible outcome in a clinical situation.

This study was aimed to primarily determine whether n-3 LCPUFA and ibuprofen were suitable candidates for use as an adjunct therapy in TB treatment, as well as a possible target for host HDTs. Even though the potential benefits of ibuprofen and n-3 LCPUFAs have been demonstrated in TB treatment, our study is the first to our knowledge to have administered the ibuprofen or n-3 LCPUFAs together with standard TB antibiotics, and these effects being assessed at different time points. This study was also aimed at contributing to preclinical data needed to precede human studies, providing evidence of whether there are any interactions between the standard TB antibiotics and a therapeutic dose of n-3 LCPUFAs or ibuprofen.

## Conclusion

Collectively, our results support the use of EPA/DHA supplementation during TB treatment, as it does not interfere with standard TB regimen, it functions to reduces *Mtb*-elicited immunopathology and also promote pro-resolving LM production, especially considering the long treatment duration of TB treatment. Though ibuprofen did elicit anti-inflammatory effects and protected the host by limiting TB-associated immunopathology, it may be more appropriately used transiently as an adjunct therapy, since it appeared to attenuate the effect of the TB antibiotics over the long term, and should, therefore, be used acutely and with care. Our study demonstrates that EPA/DHA as adjunct therapy reduces *Mtb* burden and suppresses TB pulmonary immunopathology in the long term. Thus, EPA/DHA and ibuprofen show promise to limit TB associated inflammation and improve other clinical outcomes. If successful in human application, these treatment options may assist to improve TB clinical outcomes in the near future.

## Data Availability Statement

The raw data supporting the conclusions of this article will be made available by the authors, without undue reservation.

## Ethics Statement

The animal study was reviewed and approved by The AnimCare Animal Research Ethics Committee of the North-West University, Potchefstroom, South Africa (ethics number: NWU-00055-19-S5), and the Animal Research Ethics Committee of the University of Cape Town, Cape Town, South Africa (FHS AEC 019_023).

## Author Contributions

LM headed the project. FH, LM, RD, RB and AN conceptualised and planned the experiments. FH, LM, MO and SP investigated and performed the experiments. FH, LM and MO analysed the data. FH, LM, MO and SP contributed to the interpretation of the results. FH took the lead in writing the manuscript. RD, FB and SP were involved in acquiring resources and funding for the experiment. All authors (LM, RD, RB, AN, DL, CS, FB, MO and SP) provided critical feedback and helped to shape the research, analysis and manuscript. All authors contributed to the article and approved the submitted version.

## Funding

This research was conducted at the UCT BSL3 facilities, supported by core funding from the Wellcome Trust (203135/Z/16/Z) and also student PhD study supported by the Nutricia Research Foundation.

## Disclaimer

The views and opinions expressed in the study are solely those of the authors, based on scientific reasoning and not that of Nutricia Research Foundation or the Wellcome Trust.

## Conflict of Interest

The authors declare that the research was conducted in the absence of any commercial or financial relationships that could be construed as a potential conflict of interest.
