# Supplementary figures and images for: Longer-Term Omega-3 LCPUFA More Effective Adjunct Therapy for Tuberculosis Than Ibuprofen in a C3HeB/FeJ Tuberculosis Mouse Model
Source: Front Immunol. 2021 Apr 28;12:659943. doi: 10.3389/fimmu.2021.659943 (PMC8113969; doi:10.3389/fimmu.2021.659943)

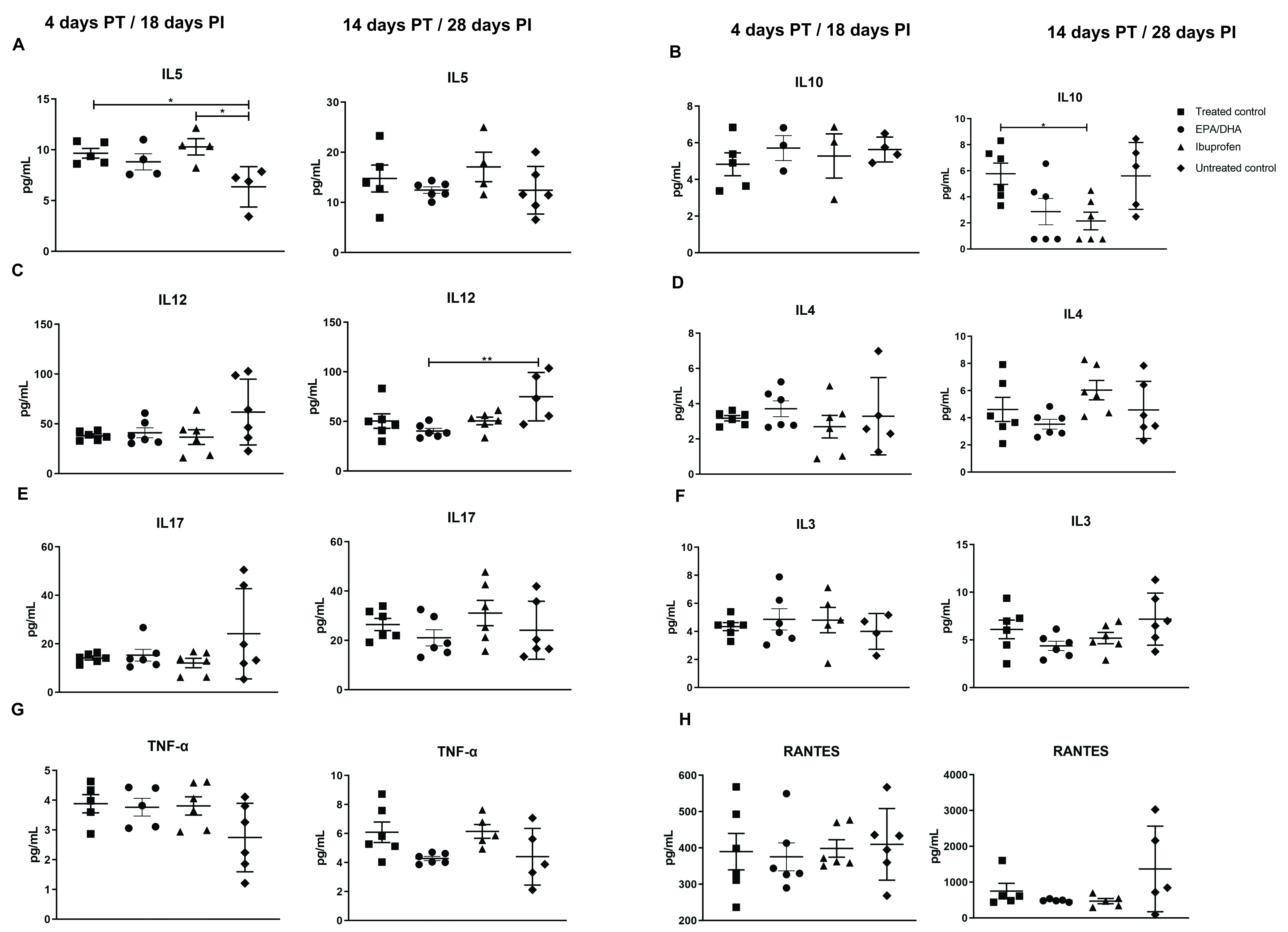

Supplement: Supplementary file 1 [file Image_1.jpeg]

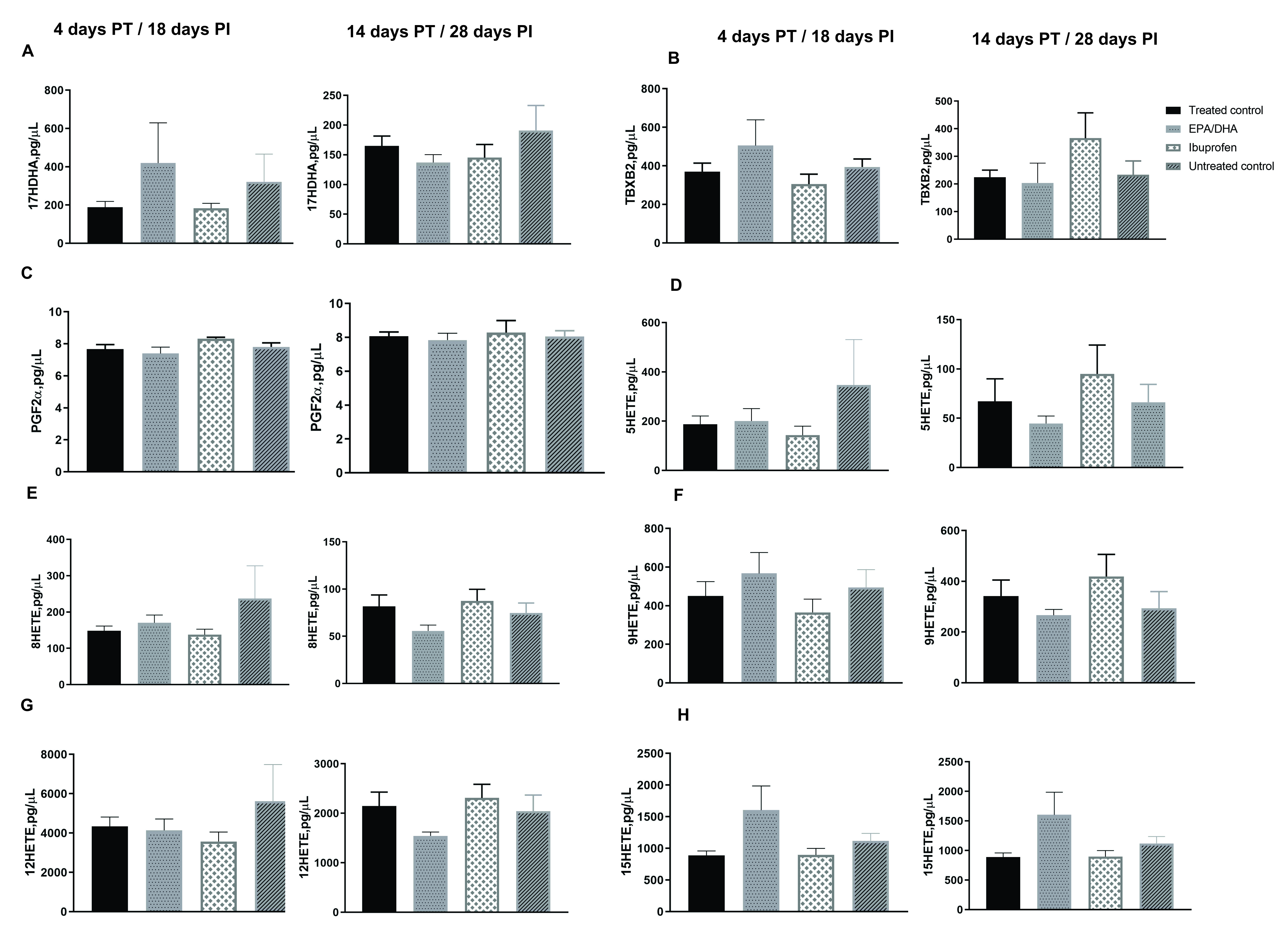

Supplement: Supplementary file 2 [file Image_2.jpeg]
