# Supplementary material for: Longer-Term Omega-3 LCPUFA More Effective Adjunct Therapy for Tuberculosis Than Ibuprofen in a C3HeB/FeJ Tuberculosis Mouse Model
Source: Front Immunol. 2021 Apr 28;12:659943. doi: 10.3389/fimmu.2021.659943 (PMC8113969; doi:10.3389/fimmu.2021.659943)
Supplement: Supplementary file 3 [file Table_1.docx]

Supplementary Materials

Supplementary file 1

***Detailed description of how the experiment was conducted***

Using mice, a mammalian model known to predict human treatment responses, and selecting conservative human bioequivalent doses, we tested n-3 LCPUFA and/or ibuprofen as adjunctive therapy together with standard TB medication. A total of 48 C3HeB/FeJ mice, between post-natal 10-12 weeks old were conditioned on a standardised AIN-93G (soybean oil at 70 g/kg diet and hydrogenated coconut oil at 30 g/kg diet and 8 g Fe/kg (40 ppm Fe)) purified rodent diet for six weeks before infection. The mice were then infected with H37Rv strain of *Mtb* by means of aerosol infection with a total lung bacterial load ~50-70 CFU. One-day post TB infection (PI day 1), 4 mice were euthanized to measure infection level in the lungs to confirm the establishment of infection. Two weeks (14 days) after infection, the mice which were kept under the same environmental conditions, were randomly allocated to four treatment groups. The first group which were the treated control group received Rifafour^®^ only (150 mg rifampicin + 75 mg isoniazid + 400 mg pyrazinamide + 275 mg ethambutol) for 3 days (n = 12) in the first phase, followed by rifampicin and isoniazid for 11 days (n = 6) in the second phase of the experiment. The second group, the EPA/DHA group received an EPA/DHA-enriched diet plus Rifafour^®^ for 3 days (n = 12) in the first phase, followed by the EPA/DHA-enriched diet plus rifampicin and isoniazid only for 11 days (n = 6) in the second phase of the experiment. The EPA/DHA-supplemented diet consisted of an AIN-93 standard diet enriched with Incromega oil (Incromega TG4030 oil DHA 500 TG SR (minimum 44% of FA as EPA; minimum 28% of FA as DHA)), which was administered *ad libitum* throughout the experiment. The third group, the ibuprofen group received ibuprofen plus Rifafour^®^ for 3 days (n = 12) in the first phase, followed by ibuprofen plus rifampicin and isoniazid for 11 days (n = 6), in the second phase of the experiment. The untreated control group, which was the fourth group received no treatment throughout the entire duration of the experiment (n = 12). In the first phase, ibuprofen and Rifafour^®^ (150 mg rifampicin + 75 mg isoniazid + 400 mg pyrazinamide + 275 mg ethambutol) were administered by oral gavage, and in the second phase, ibuprofen as well as rifampicin and isoniazid (RH) were administered in drinking water (Cornell model). At the end of each experimental phase, mice were euthanized to assess early effects and interactions. Euthanasia procedures were performed in accordance with the South African National Guidelines and University of Cape Town practice for laboratory animal standard operating procedure for euthanasia, at the P3 safety unit, by halothane exposure followed by cervical dislocation.

All drugs used for treatment were either dissolved or suspended in distilled water and administered either by oesophageal gavage or in the drinking water. The following doses were used: in phase one (intensive phase), each mouse received 0.2 mL of antibiotic consisting of Rifafour^®^ (150 mg rifampicin + 75 mg isoniazid + 400 mg pyrazinamide + 275 mg ethambutol) dissolved in 30 ml distilled water through oral gavage administration. In phase two (continuation phase), isoniazid (0.1g/L) and rifampicin (0.1g/L) were delivered to mice in drinking water. For the adjunct ibuprofen group, 0.05 g/L of ibuprofen (Nurofen^®^ cherry flavour, purchased from local pharmacy) was administered *ad libitum* in phases one and two. Because of the bitter taste of rifampicin-isoniazid together with ibuprofen, 1% sucrose was added to drinking water for three groups in phase 2. The drinking water containing the treatments were changed every three days and water consumption volume was measured in phase two to confirm equal drug intake in all three groups. For the EPA/DHA supplementation diet, intakes of a therapeutic dose of approximately 2 mg DHA and 3 mg EPA daily or not less than 1% of total energy intake when calculated on average daily food consumption was considered sufficient based on the composition of the diet.

**Supplementary figure titles**

**Supplementary figure 1**: **Treatment effects on lung cytokine/chemokines homogenate levels**. (A) IL-5, (B) IL-10, (C) IL-12, (D) IL-4, (E) IL-17, (F) IL-3, (G) TNF-α, and (H) RANTES. All data are presented in pg/mL. All mice except untreated controls were on standard TB antibiotics Rifafour^®^ for 4 days of treatment, then rifampicin and isoniazid (RH) for 10 days. All values represent mean ± SEM. Results repeated in two experiments, data shown for one experiment (n = 6 per group). One-way ANOVA followed by Tukey’s post-hoc test was used to compare means, *P < 0.05, **P < 0.01, ***P < 0.001. EPA, eicosapentaenoic acid; DHA, docosahexaenoic acid; IL, interleukin; TNF- α, tumor necrosis α; RANTES, regulated on activation, normal T cell expressed and secreted; PT, post treatment; PI, post infection

**Supplementary figure 2**: **Treatment effects of lipid mediators in crude lung homogenate at the local site of intervention**. (A) 17-HDHA, (B)TBXB2, (C) PGF2 α, (D) 5-HETE, (E) 8-HETE, (F) 9-HETE, (G) 12-HETE, and H) 15-HETE. All data are presented in pg/µL. All mice except untreated controls were on standard TB antibiotics Rifafour^®^ for 4 days of treatment, then rifampicin and isoniazid (RH) for 10 days. All values represent mean ± SEM. Results repeated in two experiments, data shown for one experiment (n=6 per group). One-way ANOVA followed by Tukey’s post-hoc test was used to compare means, *P < 0.05, **P < 0.01 and ***P < 0.001. EPA, eicosapentaenoic acid; DHA, docosahexaenoic acid; IL, interleukin; HETE, Hydroxyeicosatetraenoic acid; 17-HDHA, 17-hydroxydocosahexaenoic acid; PGF2 α, prostaglandin F2α; TBXB2, thromboxane B2; PT, post treatment; PI, post infection
